# Chronic Constrictive Pericarditis in Northeast India: A 10-Year Single-Center Study of Demographic and Clinical Profiles

**DOI:** 10.7759/cureus.72953

**Published:** 2024-11-03

**Authors:** Rajeev Bharadwaj, Reuben L Kynta, Sanjib Rawat, Bifica Lyngdoh

**Affiliations:** 1 Department of Cardiology, All India Institute of Medical Sciences, Guwahati, Guwahati, IND; 2 Department of Cardiothoracic and Vascular Surgery, North Eastern Indira Gandhi Regional Institute of Health and Medical Sciences, Shillong, IND; 3 Department of Cardiothoracic and Vascular Surgery, All India Institute of Medical Sciences, Guwahati, Guwahati, IND; 4 Department of Pathology, All India Institute of Medical Sciences, Guwahati, Guwahati, IND

**Keywords:** chronic constrictive pericarditis, demographic pattern, extra-pulmonary tuberculosis (eptb), north-east india, rapid review

## Abstract

Introduction

Chronic constrictive pericarditis (CCP) is a progressive disease characterized by thickening and fibrosis of the pericardium, leading to restricted diastolic filling and impaired cardiac function. In Northeast India, limited healthcare infrastructure poses challenges in diagnosing and managing CCP, potentially worsening patient outcomes. Therefore, this study aims to assess the demographic and clinical profiles of patients with CCP in this region over 10 years and compare our findings to the relevant but limited published literature in South Asia.

Methods

We retrospectively analyzed the medical records of 42 patients diagnosed with CCP referred to our department from 2011 to 2020. Demographic, clinical, and paraclinical data during hospitalization and follow-up were collected. All patients underwent clinical examination imaging studies, including high-resolution chest computed tomography and echocardiography. Symptomatic patients underwent open pericardiectomy, and postoperative histopathological examination was performed.

Results

Of the 42 patients, 34 (81%) were male and eight (19%) were female. A significant proportion of the patients were under the age of 20 years (n = 18, 42.9%), followed by the 21- to 40-year age group (n = 13, 31%). Eighteen patients (42.9%) were from Meghalaya, 12 (28.6%) were from Assam, and seven (16.7%) were from Nagaland. Twenty-two patients (52.4%) had a prior history of pulmonary or extrapulmonary tuberculosis (TB) and had received antitubercular therapy for at least six months.

Dyspnea on exertion was the most common symptom among the patients: 12 (28.6%) of them were classified as New York Heart Association class IV, 20 (47.6%) as class III, and 10 (23.8%) as class II. Clinical examination revealed pedal edema in 36 (85.7%), hepatomegaly in 22 (52.4%), ascites in 15 (35.7%), and pleural effusion in 13 (31%) patients. Echocardiography showed septal bounce in 26 (61.9%), pericardial calcification in 17 (40.5%), and hepatic vein flow reversal in 12 (28.6%) patients. All patients were on diuretics and digoxin before surgery. Postoperative biopsy confirmed TB as the etiology in 28 (66.7%) patients and nonspecific etiology in 14 (33.3%) of them.

Conclusions

CCP in Northeast India predominantly affects young males and is largely associated with TB. Despite efforts to control TB, it remains a major contributor to CCP in this region. Recognizing the clinical presentation and diagnostic profile is essential for improving management strategies and patient outcomes.

## Introduction

Chronic constrictive pericarditis (CCP) is a progressive disease characterized by thickening and fibrosis of the pericardium, which restricts diastolic filling and impairs cardiac function. CCP can result from various etiologies, including infections - particularly tuberculosis (TB) - previous cardiac surgery, radiation therapy, and idiopathic causes. The rigid pericardium limits the normal expansion of the heart during diastole, leading to increased venous pressure, decreased cardiac output, and eventual heart failure symptoms [1].

In Northeast India, the healthcare infrastructure poses significant challenges in diagnosing and managing CCP. Limited access to advanced diagnostic tools, such as echocardiography, can delay diagnosis and appropriate management, leading to worse outcomes. Moreover, specialized cardiac surgical centers are limited, necessitating referral to tertiary care centers outside the state for definitive surgical interventions like pericardiectomy. Therefore, this study aims to describe the demographic and clinical profiles of patients with CCP in Northeast India over 10 years, identify the predominant etiologies, and highlight the challenges in diagnosis and management specific to this region.

## Materials and methods

We retrospectively analyzed the medical records of patients with CCP referred to our department for further management from 2011 to 2020. A total of 42 patients met the criteria for CCP. Demographic variables were recorded, and clinical and paraclinical data during hospitalization and follow-up were collected. Informed consent was obtained from all the participants in the study. The North Eastern Indira Gandhi Regional Institute of Health & Medical Science (NEIGRIHMS) Scientific Advisory Committee (NSAC) Institute Ethics Committee (IEC) (Human Studies) issued the approval for the study. 

All patients underwent clinical examination along with sonographic and radiologic evaluations, including high-resolution chest computed tomography. Patients with documented pathogenic infections received antibiotic therapy according to culture sensitivity before primary or secondary surgical intervention. All patients underwent surgical intervention in the form of open pericardiectomy.

Statistical analysis

Continuous variables are expressed as mean ± standard deviation and categorical variables as percentages. We used analysis of variance or the Mann-Whitney test for quantitative variables, and Fisher’s exact test for qualitative variables. A p-value less than 0.05 was considered statistically significant.

## Results

We identified 42 patients diagnosed with CCP between 2011 and 2020. Of these, 34 (81%) were male and eight (19%) were female. A large proportion of patients were under 20 years of age (n = 18, 42.9%), with the second-largest age group being 21-40 years (n = 13, 31%). Eighteen (42.9%) patients were from Meghalaya, 12 (28.6%) from Assam, and seven (12.7%) from Nagaland. Twenty-two (52.4%) patients had a prior history of pulmonary or extra-pulmonary TB and had taken antitubercular therapy (for at least six months). The demographic and clinical profile details are presented in Tables 1, 2.

**Table 1:** Demographic and clinical profile of the patient population ATT: antitubercular therapy, DOE: dyspnea on exertion, NYHA: New York Heart Association, JVP: jugular venous pressure.

Demographic Data			N (%)
Sex		Male	34 (81%)
		Female	8 (19%)
Age (years)		<20	18 (42.9%)
		21-40	13 (31.0%)
		41-60	9 (21.4%)
		>60	2 (4.8%)
Symptoms	Prior ATT	Yes	22 (52.4%)
		No	20 (47.6%)
	DOE NYHA	II	10 (23.8%)
		III	20 (47.6%)
		IV	12 (28.6%)
Signs	Elevated JVP	Yes	29 (69%)
		No	13 (31%)
	Hepatomegaly	Yes	22 (52.4%)
		No	20 (47.6%)
	Pleural effusion	Yes	13 (31%)
		No	29 (69%)
	Ascites	Yes	15 (35.7%)
		No	27 (64.3%)
	Pedal edema	Yes	36 (85.7%)
		No	6 (14.3%)
Echocardiography	Septal bounce	Yes	26 (61.9%)
		No	16 (38.1%)
	Calcification	Yes	17 (40.5%)
		No	25 (59.5%)
	Hepatic vein flow reversal	Yes	12 (28.6%)
		No	30 (71.4%)

**Table 2:** Location of the study population NA: not applicable.

Location	N	Percent	Cumulative percent
Meghalaya	18	42.90%	42.90%
Assam	12	28.60%	71.40%
Nagaland	7	16.70%	88.10%
Mizoram	1	2.40%	90.50%
Manipur	1	2.40%	92.90%
Others	3	7.10%	100.00%
Total	42	100.00%	NA

Dyspnea on exertion was the most common symptom, with 12 (28.6%) patients exhibiting New York Heart Association class IV symptoms, and 20 (47.6%) and 10 (23.8%) displaying class III and class II symptoms, respectively. On clinical examination, 22 (52.4%) patients had hepatomegaly, 36 (85.7%) had pedal edema, 15 (35.7%) presented with ascites, and 13 (31%) had pleural effusion. On echocardiography, 26 (61.9%) patients showed septal bounce and pericardial calcification was seen in 17 (40.5%) patients. Hepatic vein flow reversal was observed in 12 (28.6%) patients. All the patients were on diuretics and digoxin before surgery.

**Figure 1:**
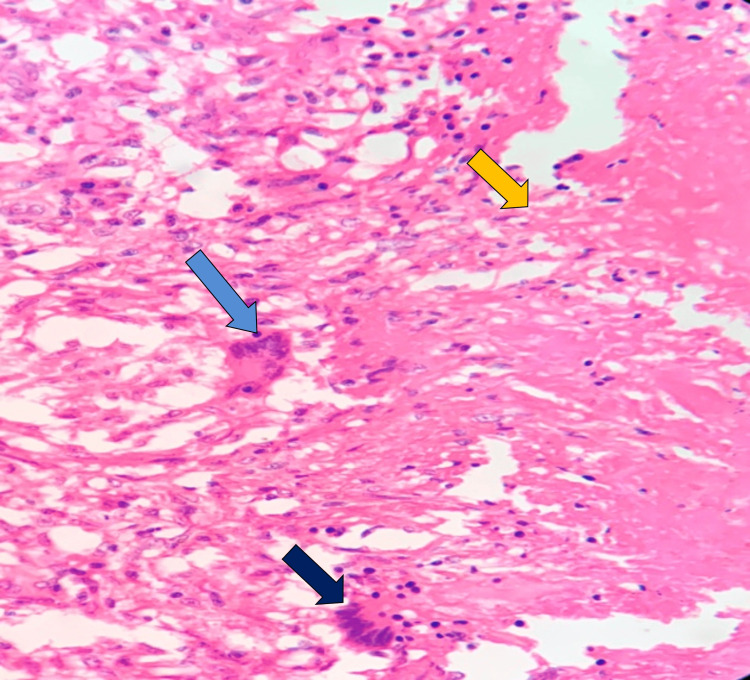
Hematoxylin and Eosin (H&E) stained sections of tuberculous pericarditis showing epithelioid granulomas (blue arrow), caseous necrosis (yellow arrow), and Langhans giant cells (black arrow)

**Figure 2:**
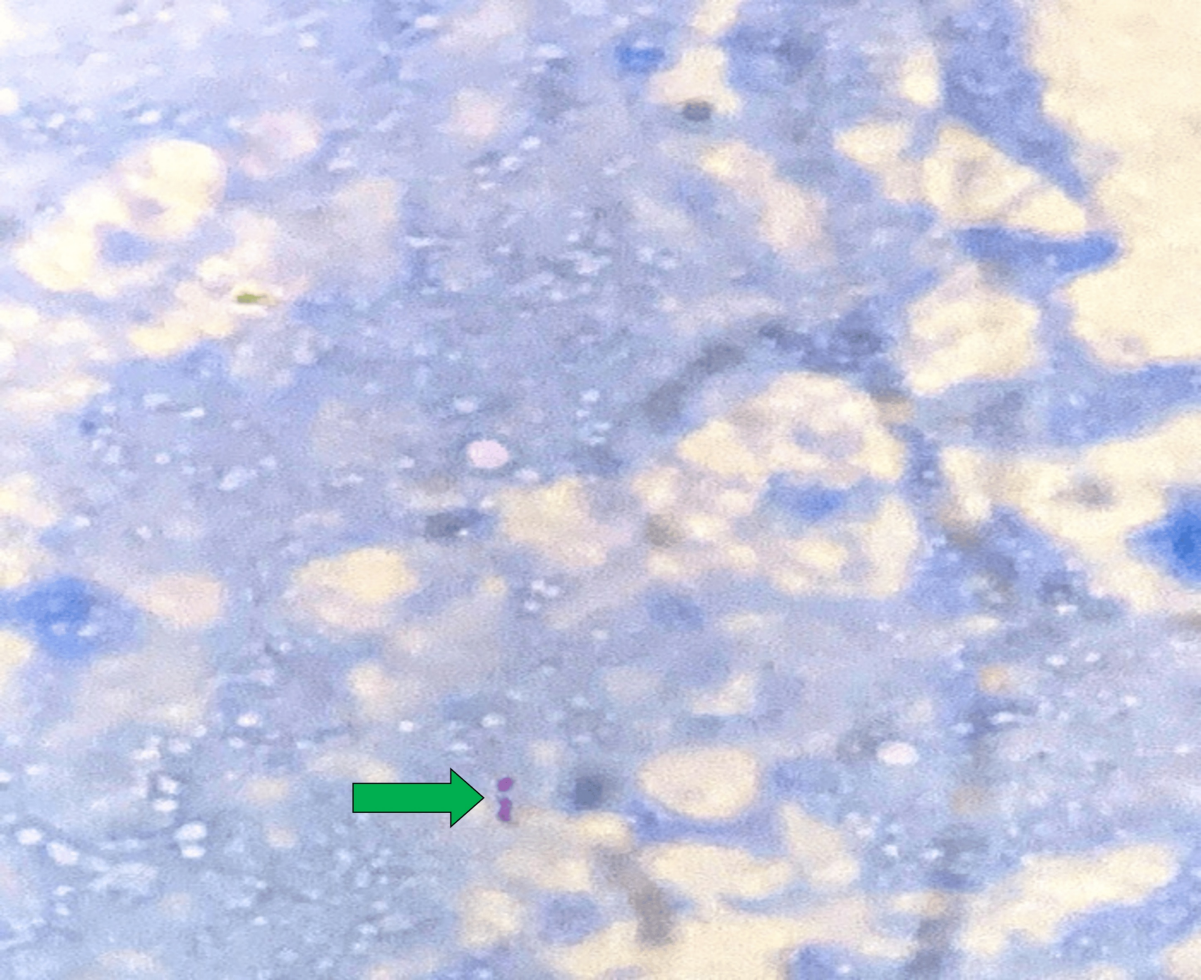
Modified Ziehl-Neelsen stain showing acid-fast bacilli (green arrow) on high-power microscopy

Post-surgical biopsy confirmed TB as the etiology in 28 (66.7%) patients with evidence of caseous necrosis, epithelioid granulomas, Langhans giant cells, and specialized staining showing acid-fast bacilli, as depicted in Figures 1, 2.

Post-surgical biopsy also revealed non-specific etiology in 14 (33.3%) patients, as shown in Table 3.

**Table 3:** Incidence of biopsy diagnoses NA: not applicable, TB: tuberculosis.

Diagnosis	Incidence (N)	Valid percent	Cumulative percent
TB	28	66.70%	66.70%
Non-specific	14	33.30%	100.00%
Total	42	100.00%	NA

## Discussion

In our study, most patients with CCP were younger than 40 years. Similar results were reported by Yangni-Angate et al., who found CCP in patients with a mean age of 28.8 ± 10.44 years [2]. However, higher mean ages were reported in studies by Lin et al. (40.1 ± 15.5 years), Ghavidel et al. (46.6 ± 14.9 years), and Karima et al. (40.46 ± 16.74 years) [3-5]. The comparatively younger age group in our study may be explained by the higher incidence of TB in the Northeast region and those contacts leading to CCP presentation at an earlier age.

We conducted a brief review comparing clinical, demographic, and etiological data of CCP patients from South Asia, as depicted in Table 4 [6-10]. Inclusion criteria for the review were studies from South Asia, particularly India. When data from India were not available, we included relevant data from neighboring countries. We included studies that had full texts available online and provided clinical and etiological data, regardless of treatment and management strategies.

**Table 4:** Comparison of the clinical and etiological profiles of CCP patients from studies in South Asia with our study participants CCP: Chronic constrictive pericarditis, JVP: Jugular venous pressure, CVP: central venous pressure, NR, not reported.

Parameter	Potlabathini et al., 2016 [6]	Patil et al., 2016 [7]	Acharya et al., 2018 [8]	Bashi et al., 1988 [9]	Benjamin et al., 2022 [10]	Our study
CCP cases, n	25	20	130	118	124	42
NYHA class data, n (%)	24 (96%) Exertional dyspnea (Class not mentioned)	17 (85%) in Class III	118 (90.77%) in Class III-IV	57 (48%) in Class III	91 (73.3%) Class II	20 (47.6%) in Class III
Pedal edema, n (%)	8 (32%)	9 (45%)	53 (40.77%)	99 (84%)	101 (81.5%)	36 (85.7%)
Ascites, n (%)	18 (75%)	18 (90%)	81 (62.31%)	106 (90%)	69(55.6%)	15 (35.7%)
Elevated JVP, n (%)	24 (96%)	20 (100%)	NR; mean CVP 20.9 mmHg	118 (100%)	77 (62%)	29 (69%)
Pleural effusion, n (%)	5 (20%)	NR	28 (21.54%)	64 (54%)	80 (64.5%)	13 (31%)
Hepatomegaly, n (%)	22 (88%)	17 (85%)	98 (75.38%)	118 (100%)	NR	22 (52.4%)
Calcification, n (%)	10 (40%)	7 (35%)	NR	25 (21%)	33 (27%)	17 (40.5%)
Septal bounce, n (%)	9 (36%)	20 (100%)	NR	NR	NR	26 (61.9%)
Hepatic vein flow reversal, n (%)	NR	NR	NR	NR	NR	12 (28.6%)
Etiology (tubercular), n (%)	14 (56%)	NR	NR	NR	64 (51.6%)	28 (66.7%)

Our study showed that constriction is more predominant in males (81%), which is consistent with previous studies [11-13]. There is no clear explanation for this finding, but the male-to-female ratio in our study is 4:1, similar to the study by Schwefer et al. (1.7 to 4:1) [14]. Our study’s demographic distribution is unique, as no archived data for Northeast India are available in research databases.

In the present study, patients were significantly sicker, with 76% of study population exhibiting New York Heart Association class III and IV symptoms, which was one of the reasons they were referred for pericardiectomy. Additionally, 52.4% of patients had a history of antitubercular therapy. TB is responsible for early or late pericardial constriction, and can present either within a few months or several years later, while in Western countries the majority of causes are post-surgical and post-radiotherapy [15,16].

On clinical examination, pedal edema (85.7%) was the most frequent finding in our study, followed by hepatomegaly (52.4%) and ascites (35.7%), which aligns with the classical presentation of pedal edema occurring before ascites in cases of CCP. Pleural effusion was the least common finding in our study (31%). The most common physical signs described in the literature are hepatomegaly (23.4%-100%), jugular venous distension (52%-65%), ascites (8.9%-90%), peripheral edema (8.9%-84%), and associated unilateral or bilateral pleural effusions (35%-79.3%) [9,17].

Diagnosis of CCP was confirmed using two-dimensional echocardiography, with the detection of septal bounce (61.9%), which is the most common finding described in the literature after a dilated inferior vena cava [18]. Hepatic vein flow reversal during expiration, being the most specific finding, was present in only 28.6% of cases in our study. Calcification, although a less common finding in Western literature, was present in 40.5% of cases in our study, possibly due to the pathological nature of chronic inflammation from TB. Postoperative biopsy of the pericardial tissue showed an etiological diagnosis of TB in 66.7% of our cases. A previous study from South Africa showed an etiological diagnosis of TB in 32.7% of cases on biopsy but suggested TB as a cause of constriction in 90% of cases by occurrence much later after active TB [19]. This shows that pericardial biopsy might not find pathological findings of tuberculous pericarditis and there might not be any acid-fast bacilli detected to label the cause as tubercular from biopsy alone. Only after the occurrence of active TB, in any pulmonary or extra-pulmonary form, can a retrospective causation of constriction be attributed to TB. 

Limitations

Our study had several important limitations. As a retrospective analysis, it is subject to the inherent biases associated with such study designs, including potential inaccuracies and incomplete documentation in the medical records. The study was conducted at a single tertiary care center, which may limit the generalizability of our findings to other regions or healthcare settings. Also, the small sample size over the 10-year period reduces the study’s statistical power and may limit the detection of significant associations or trends. We did not include a control group or comparative cohort, which restricts our ability to draw definitive conclusions about the causal relationship between TB and CCP. The lack of long-term follow-up data prevents us from assessing the patients' post-operative outcomes and survival rates, limiting our understanding of the long-term efficacy of pericardiectomy in this population. Lastly, we did not account for potential confounding factors like socioeconomic status, access to healthcare services, or comorbid conditions, which could have influenced both the presentation and management of CCP in our patient population.

## Conclusions

Our study attempted to describe CCP from a tertiary care center experience in Northeast India, highlighting the regional patterns and distinctive clinical features. Our findings revealed that CCP commonly affects younger individuals, with a predominance in males. The disease presentation is often delayed with most patients reporting symptoms such as exertional dyspnea, abdominal distension, and peripheral edema only after a significant progression of the condition, wherein surgical intervention (pericardiectomy) is the only option available to reduce morbidity. Despite major efforts by national programs for its control and elimination, TB remains the major contributor to CCP. It remains a prominent etiological agent in the region, underscoring the continued impact of infectious diseases on CCP pathology. This clinical presentation, diagnostic, and etiological profile will help further improve the management strategies of this rare entity.
